# Regulatory Role of *Nfix* Gene in Sheep Skeletal Muscle Cell Development and Its Interaction Mechanism with *MSTN*

**DOI:** 10.3390/ijms252211988

**Published:** 2024-11-08

**Authors:** Meiyu Qiu, Xuemei Zhang, Li Liao, Ning Zhang, Mingjun Liu

**Affiliations:** 1 Institute of Biotechnology, Xinjiang Academy of Animal Science, Xinjiang Key Laboratory of Animal Biotechnology, Urumqi 830026, China; 2 College of Animal Science, Xinjiang Agricultural University, Urumqi 830052, China

**Keywords:** *Nfix*, temporal analysis, *MSTN*, miRNA, skeletal muscle cells

## Abstract

Skeletal muscle development is crucial for livestock production, and understanding the molecular mechanisms involved is essential for enhancing muscle growth in sheep. This study aimed to investigate the role of *Nfix*, a member of the nuclear factor I (NFI) family, in regulating muscle development in sheep, filling a significant gap in the current understanding of *Nfix* deficiency and its impact on skeletal muscle growth, as no similar studies have been reported in this species. Bioinformatic analysis, including temporal analysis of transcriptome data, identified *Nfix* as a potential target gene for muscle growth regulation. The effects of *Nfix* overexpression and knockout on the proliferation and differentiation of sheep skeletal muscle cells were investigated. Changes in the expression of associated marker genes were assessed to explore the regulatory link between *Nfix* and the myostatin (*MSTN*) gene. Additionally, target miRNAs for *Nfix* and *MSTN* were predicted using online databases such as miRWalk, resulting in the construction of an *Nfix*–miRNA–*MSTN* interactive regulatory network. The findings revealed that *Nfix* promotes the proliferation and differentiation of sheep skeletal muscle cells, with further analysis indicating that *Nfix* may regulate muscle cell development by modulating *MSTN* expression. This study provides preliminary insights into the function of *Nfix* in sheep skeletal muscle development and its regulatory interactions, addressing a critical knowledge gap regarding *Nfix* deficiency and its implications for muscle growth. These findings contribute to a better understanding of muscle biology in sheep and provide a theoretical foundation for future research into the regulatory mechanisms governing muscle development.

## 1. Introduction

In vertebrates, muscle progenitor cells originate from the mesoderm, differentiating from myotome and dermatomyotome cells [1,2,3]. The formation of skeletal muscle fibers typically involves the development of primary and secondary fibers, with some species exhibiting a third stage that gives rise to tertiary fibers [4,5,6]. In livestock, muscle mass increases through two primary pathways: the proliferation of muscle fibers and the hypertrophy of existing fibers. Before birth, the number of skeletal muscle fibers increases, accompanied by some degree of hypertrophy. After birth, the fiber count generally remains constant, and muscle growth primarily results from the increase in fiber diameter. Therefore, the embryonic period is critical for skeletal muscle development, as the number of fibers determines the muscle’s growth potential after birth.

Key regulatory factors play essential roles in this developmental process. Among these, the transcription factor *Nfix*, a member of the nuclear factor I (NFI) family, has emerged as a crucial player in muscle fiber formation. The NFI family is a group of DNA-binding proteins with specific binding sites [7], also known as CTF or CAAT box transcription factor, playing a significant role in viral DNA replication and gene expression regulation. The NFI family consists of four members—*NFIA*, *NFIB*, *NFIC*, and *NFIX*—characterized by their conserved N-terminal DNA-binding domains. NFI proteins form dimers with double-stranded DNA by binding to the palindrome sequence TTGGC(N5)GCCAA or its variants [8,9]. These interactions regulate gene transcription, as NFI-binding sites are present in the promoters of muscle differentiation-related genes [10,11]. NFI proteins can bind to these promoters to regulate gene expression [12]. *Nfix* activates fetal muscle-specific genes like *MCK* by binding to protein kinase Cu (PKCu) and myocyte enhancer factor 2A (*Mef2A*), while also inhibiting the embryonic gene *MyHC-I*. *Nfix*-deficient mice show reduced body size and developmental defects. Additionally, lacking *Nfix* slows muscle regeneration after injury and increases oxidative slow-twitch fibers, which are more resilient to oxidative stress. During development in mice and zebrafish, *Nfix* counteracts *Sox6*, facilitating proper *MyHC* expression [13,14].

Concurrent with the activity of *Nfix*, *MSTN*, a member of the TGF-β superfamily is a major negative regulator of muscle growth. *MSTN* inhibits myocyte differentiation by downregulating key myogenic regulatory factors, such as *MyoD* and *Myogenin*, thus exerting a powerful influence on muscle fiber formation [15]. The addition of TGFβ1 significantly reduces the number of nuclei in newly formed muscle fibers, leading to a decreased cross-sectional area. Exogenous TGFβ can impair muscle function during regeneration, while inhibiting *TGFβR2* promotes larger muscle fibers [16]. *MSTN* negatively regulates muscle size, with loss-of-function mutations increasing muscle mass across species, including humans [17]. *MSTN* inhibits myocyte differentiation by downregulating *MyoD* and *Myogenin* and reducing creatine kinase activity [18]. *MyoD* is a downstream target of *Smad3*, which *MSTN* activates to inhibit *MyoD* transcription [19,20]. *MSTN* also upregulates *p21* and reduces *Cdk2* activity in muscle satellite cells, preventing proliferation and maintaining quiescence [21]. In Texel sheep, a G→A mutation in the *MSTN* 3′UTR increases miR206 and miR1 expression, inhibiting *MSTN* transcription and resulting in double-muscling traits [22].

Numerous studies have shown that miRNA is essential for the proliferation and differentiation of skeletal muscle cells across species by targeting the 3′UTR of specific genes. In mice, miR-143 negatively regulates *IGFBP5* during muscle development [23]. In poultry, miR-21-5p influences *KLF3* in chicken satellite cells, while miR-320-3p targets *CFL2* for actin remodeling [24,25]. In ruminants, miR-377 inhibits the proliferation of bovine muscle satellite cells by regulating *FHL2* [26], whereas miR-27b promotes sheep muscle satellite cell proliferation by targeting *MSTN* [27].

Despite the substantial body of research on *Nfix* and *MSTN* in model organisms, their specific roles and mechanisms in sheep muscle development remain inadequately characterized. This study aimed to bridge this knowledge gap by investigating the regulatory role of *Nfix* in the proliferation and differentiation of sheep skeletal muscle cells, as well as its interaction with *MSTN*. We employed gene overexpression and knockout techniques to elucidate the effects of *Nfix* on *MSTN* expression and its downstream targets, including *MyoD* and *SMAD4*, utilizing quantitative RT-PCR and Western blotting methods. Furthermore, this research predicts the target miRNAs of *Nfix* and *MSTN* using online databases like miRWalk, allowing for the construction of an *Nfix*–miRNA–*MSTN* interaction regulatory network. This study not only seeks to enhance our understanding of the molecular mechanisms underlying muscle development in sheep but also lays the groundwork for future investigations into muscle growth regulation in livestock, ultimately contributing to improved agricultural practices and animal breeding strategies.

## 2. Results

### 2.1. Temporal Analysis Identifies Nfix as a Potential Target Gene for the Regulation of Skeletal Muscle Development

**Figure 1:**
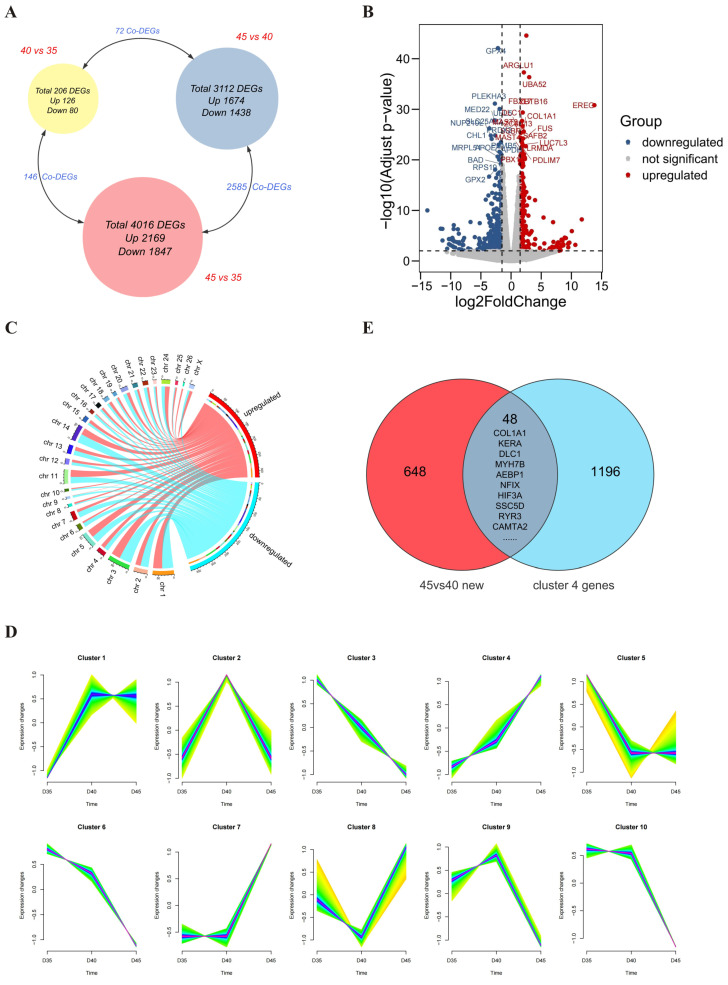
Temporal analysis screens of potential target genes related to skeletal muscle development. (**A**) Schematic diagram of differential analysis of embryo muscle sample transcriptome data. (**B**) Volcano plot of re-analyzed differential genes in 45vs40 group. (**C**) Chromosome distribution of re-analyzed differential genes in 45vs40 group. (**D**) Temporal analysis to screen continuously upregulated protein-coding genes. (**E**) Venn diagram to screen 48 continuously upregulated differential protein-coding genes.

In preliminary research results, transcriptome sequencing analysis was conducted on embryonic muscle samples taken at 35, 40, and 45 days of pregnancy (unpublished). The 40vs35, 45vs40, and 45vs35 groups identified 206, 3112, and 4016 differential genes, respectively (padj < 0.05 and absolute value of log2 fold change ≥1), showing that the number of differential genes in the 45vs40 group was significantly more than that in the 40vs35 group. This result suggests that 40–45 days may be a critical period for early muscle development in sheep (Figure 1A). To further screen the core target genes affecting early muscle development in sheep, this study re-analyzed the differential genes in the 45vs40 group. The new screening threshold was padj < 0.01 and absolute value of log2 fold change ≥1.5, resulting in 696 differentially expressed protein-coding genes, of which 324 were upregulated and 372 downregulated (Figure 1B, Table S1 (gene list)). These differential genes were mainly distributed on chromosomes 1 and 3 (Figure 1C). At the same time, we carried out temporal analysis on the muscle transcriptome data from the entire 35–40–45 developmental process and obtained 1244 continuously upregulated protein-coding genes (cluster 4) (Figure 1D, Table S2 (cluster 4 gene list)). The intersection of the above 696 differentially expressed genes in the 45vs40 group and the continuously upregulated genes in the temporal analysis results yielded 48 continuously upregulated differential protein-coding genes (Figure 1E). By sorting them according to the FPKM values, we finally obtained the top 10 potential target genes with relatively high expression levels (Table 1).

### 2.2. Construction and Transfection of Nfix Overexpression/Knockout Vectors in Sheep

**Figure 2:**
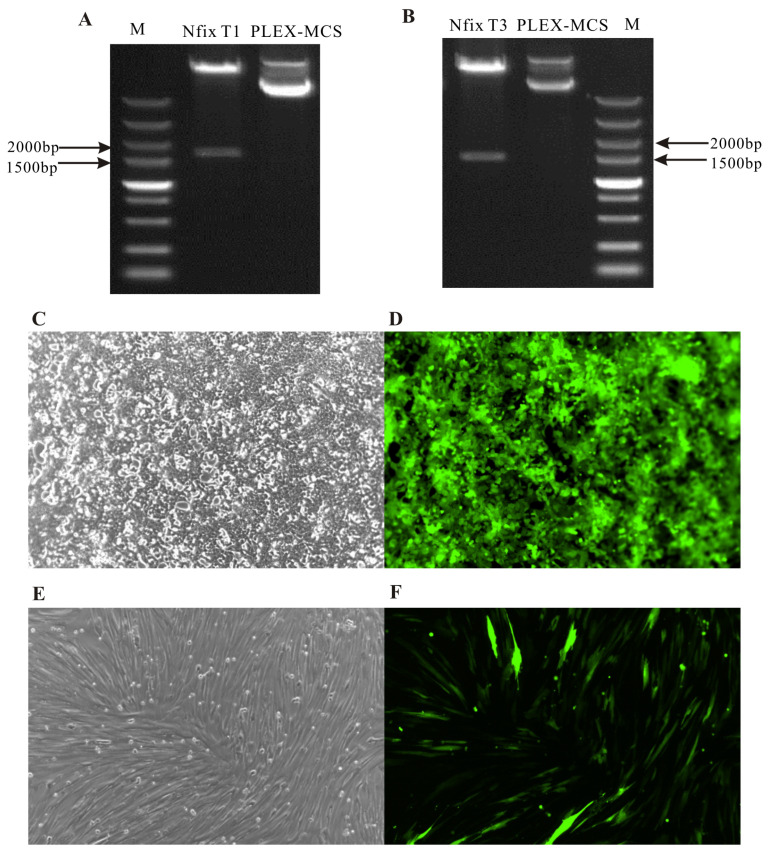
Lentivirus infection of 293T and sheep myoblast cells. (**A**) Plex-*Nfix*T1 plasmid. (**B**) Plex-*Nfix*T3 plasmid. (**C**) Plex-GFP virus infection of 293T cells under bright field. (**D**) Plex-GFP virus infection of 293T cells under FITC condition. (**E**) Plex-GFP virus infection of sheep myoblast cells under bright field. (**F**) Plex-GFP virus infection of sheep myoblast cells under FITC condition. The scales are all 100 µm.

Amplification was performed using the cDNA of a Chinese merino sheep’s longissimus dorsi muscle as a template. The target product was connected to the Plex-MCS vector and electrophoresed after enzyme digestion. The results showed bright and clear bands (Figure 2A,B), with the fragment size consistent with expectations. After sequencing the amplified products, two forms of coding sequences for the sheep *Nfix* gene were obtained, named *Nfix*T1/T3. The knockout sgRNAs were also linked to lentiCRISPRv2-Puro and sequenced. The results indicated that the sequence was consistent with the target sequence and could be used for subsequent experiments (Supplementary Materials S1). The recombinant lentiviral plasmids *Nfix*T1, *Nfix*T3, *Nfix*-sg1, *Nfix*-sg2, *Nfix*-sg3, and Plex-GFP (positive control) were co-transfected into 293T cells with packaging plasmids (psPAX2 and pMD2.G) using liposome transfection. Viruses were collected and used to infect 293T cells and sheep myoblasts. Observations under a fluorescence microscope revealed significant green fluorescence, indicating successful transfection (Figure 2C–F).

### 2.3. Nfix Promotes Myoblast Proliferation

**Figure 3:**
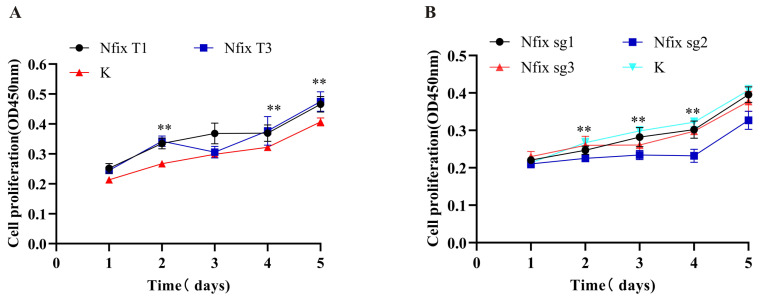
*Nfix* promotes myoblast proliferation. (**A**) CCK-8 cell proliferation assay after overexpression of *Nfix*. (**B**) CCK-8 cell proliferation assay after knockout of *Nfix*. Statistical differences between groups were determined using two-way ANOVA, and a *p*-value < 0.05 was considered statistically significant (** *p* < 0.01). K indicates a negative control.

We conducted overexpression and knockout experiments to assess the impact of *Nfix* on myoblast proliferation. Vectors for *Nfix* overexpression and knockout were constructed and transfected separately into sheep myoblasts cultured in growth medium (GM). In subsequent experiments, the knockout efficiency of *Nfix*-sg2 was found to be higher. CCK-8 assay results showed that overexpression of *Nfix* significantly increased cell proliferation 48, 96, or 120 h after transfection (Figure 3A). In contrast, *Nfix* knockout significantly inhibited the proliferation ability of sheep myoblasts compared to the negative control group (Figure 3B).

### 2.4. Nfix Positively Regulates Myogenic Differentiation

**Figure 4:**
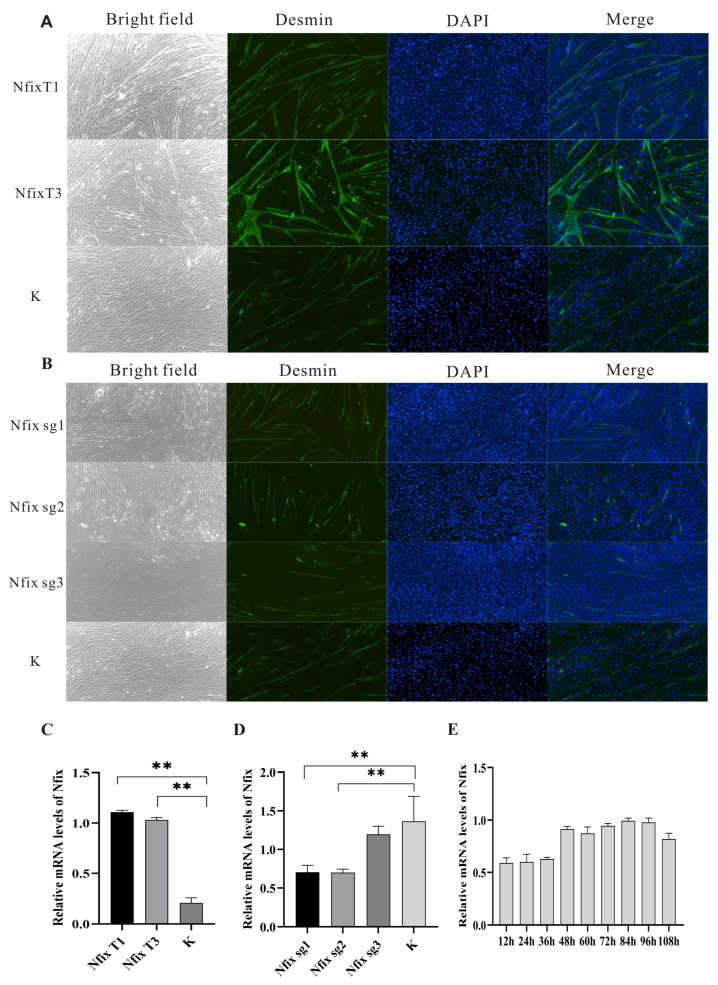
*Nfix* improved cell differentiation. (**A**,**B**) Immunofluorescence staining for desmin protein in *Nfix*-overexpressing or *Nfix*-knockout-treated myoblasts that were cultured for five days in differentiation medium. Desmin and the nucleus are stained in green and blue (DAPl), respectively. (**C**) The expression of genes after overexpression of *Nfix*. (**D**) Expression of genes after knockout of *Nfix*. (**E**) Expression of *Nfix* at different time points. Statistical differences between groups were determined using one-way ANOVA, and a *p*-value < 0.05 was considered statistically significant ** *p* < 0.01). K indicates a negative control. The scales are all 100 µm.

During the differentiation process of sheep myoblasts, we monitored the temporal changes in the expression of myogenesis-related genes and the *Nfix* gene. *Nfix* was upregulated during the myogenesis differentiation process of sheep myoblasts, which is consistent with the expression level changes in the myogenesis marker desmin (Figure 4E). Immunofluorescence staining and qRT-PCR confirmed that more myotubes were formed after *Nfix* overexpression compared to cells transfected with control vector (Figure 4A,C). Conversely, fewer myotubes were formed after *Nfix* knockout compared to cells transfected with control vector (Figure 4B,D). Overexpression of *Nfix* significantly increased the levels of myogenic markers in sheep myoblasts.

### 2.5. Nfix Regulates Myostatin Expression in Differentiating Myoblasts

**Figure 5:**
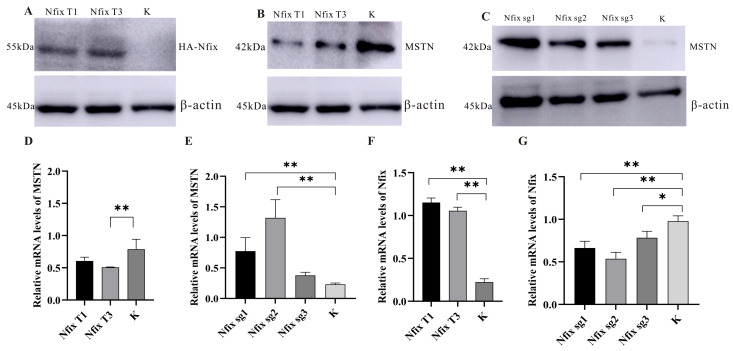
*Nfix* regulates *MSTN* expression in differentiating myoblasts. (**A**) Western blotting for HA protein in *Nfix* overexpression. (**B**) Western blotting for MSTN protein in *Nfix* overexpression. (**C**) Western blotting for MSTN protein in *Nfix* knockout. (**D**) The expression of genes after overexpression of *Nfix*. (**E**) The expression of genes after knockout of *Nfix*. (**F**) The expression of genes after overexpression of *MSTN*. (**G**) The expression of genes after knockout of *MSTN*. Statistical differences between groups were determined using one-way ANOVA, and a *p*-value < 0.05 was considered statistically significant (* *p* < 0.05, ** *p* < 0.01). K indicates a negative control. β-actin was used to normalize.

Studies have found that mouse *Nfix* can inhibit the expression of *MSTN*, and when *Nfix* is deficient, muscle regeneration is significantly delayed. This suggests that *Nfix* may affect the growth and development of mouse skeletal muscle through the regulation of *MSTN* [28]. Western blotting and qRT-PCR tests confirmed that sheep myoblasts were transduced with lentiviral vectors carrying *Nfix* overexpression (Plex-*Nfix*) or negative control (K). The expression of *Nfix* significantly increased in sheep myoblasts treated with plex-*Nfix* T1/T3 (Figure 5A). Importantly, the expression of *MSTN* was upregulated in myotubes where *Nfix* was knocked out, while the opposite was true for overexpressed *Nfix*. This confirmed that *Nfix* can downregulate the expression of *MSTN* in differentiating sheep myoblasts (Figure 5B–G).

### 2.6. Detection of the Expression of Differentiation-Related Genes After Nfix Overexpression/Knockout

**Figure 6:**
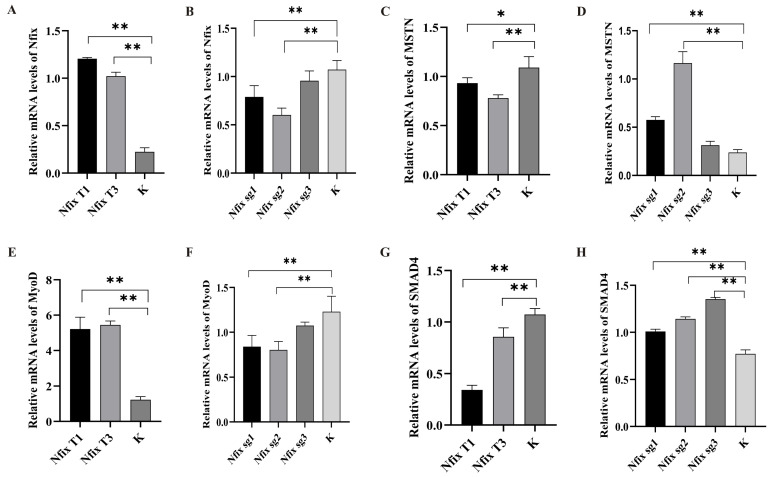
The expression of regulatory genes for the induction and differentiation into myocytes. (**A**) The expression of genes after overexpression of *Nfix*. (**B**) The expression of genes after knockout of *Nfix*. (**C**) The expression of genes after overexpression of *MSTN*. (**D**) The expression of genes after knockout of *MSTN*. (**E**) The expression of genes after overexpression of *MyoD*. (**F**) The expression of genes after knockout of *MyoD*. (**G**) The expression of genes after overexpression of *SMAD4*. (**H**) The expression of genes after knockout of *SMAD4*. Statistical differences between groups were determined using one-way ANOVA, and a *p*-value < 0.05 was considered statistically significant (* *p* < 0.05, ** *p* < 0.01). K indicates a negative control.

MS*TN* primarily plays a role in the TGF-β signaling pathway, inhibiting the proliferation and differentiation of muscle cells by transmitting signals through *Smad3* and *SMAD4*. *MyoD* is a muscle-specific transcription factor. Although it does not directly belong to the TGF-β signaling pathway, it promotes the differentiation of muscle cells by inhibiting this pathway. *Nfix* is a nuclear transcription factor that regulates the differentiation of muscle cells, especially in the late stages of differentiation. Overexpression or knockout of the *Nfix* gene significantly affects the differentiation of sheep myoblasts. To reveal its mechanism, the expression of *MSTN*, *MyoD*, *Nfix*, and *SMAD4* was detected by qRT-PCR. The results showed that after the differentiation of sheep myoblasts, the relative expression levels of *MyoD* and *Nfix* genes in non-transformed sheep myoblasts were significantly higher than those in *Nfix*-overexpressing myoblasts (*p* < 0.01), while the relative expression levels of *MSTN* and *SMAD4* genes in *Nfix*-overexpressing myoblasts were significantly lower than those in non-transformed cells (*p* < 0.05, *p* < 0.01). This indicates that *Nfix* gene expression can significantly upregulate the expression of *MyoD* genes. However, the knockout of the *Nfix* gene led to a significant upregulation of *MSTN* and *SMAD4* genes and a significant downregulation of *MyoD* and *Nfix* genes (Figure 6A–H). These results suggest that the *Nfix* gene promotes the differentiation of myoblasts, leading to a significant upregulation of genes such as *MyoD* and downregulation of the *SMAD4* gene. Conversely, knocking out *Nfix* expression reverses these gene expression patterns.

### 2.7. Construction of Nfix–miRNA–MSTN Interaction Regulation Network

**Figure 7:**
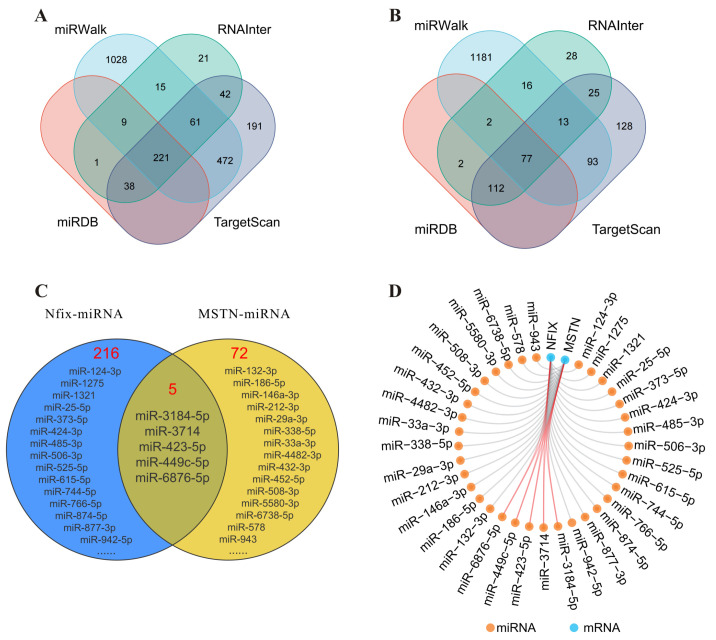
Construction of *Nfix*–miRNA–*MSTN* interaction regulation network. (**A**) Prediction of miRNAs interacting with *Nfix* in four databases. (**B**) Prediction of miRNAs interacting with *MSTN* in four databases. (**C**) Common target miRNAs of *Nfix* and *MSTN*. (**D**) *Nfix*–miRNA–*MSTN* interaction regulation network.

In order to further enrich the regulatory mechanism of the *Nfix*–*MSTN* interaction in the proliferation and differentiation process of skeletal muscle cells, a miRNA–gene interaction regulation network was constructed using online databases such as miRWalk, miRDB, RNAInter, and TargetScan. The results showed that 221 miRNAs with potential targeting relationships with *Nfix* (Figure 7A) and 77 miRNAs with potential targeting relationships with *MSTN* (Figure 7B) were predicted in the interaction network. After taking the intersection of the above miRNAs, it was found that *Nfix* and *MSTN* had five common target miRNAs (Figure 7C), among which miR-423-5p had a regulatory effect on myocyte differentiation. Therefore, we hypothesize that *Nfix* influences the proliferation and differentiation process of skeletal muscle cells by forming an interaction regulation relationship with *MSTN* through miRNA (Figure 7D). The specific regulatory mechanism will be further explored in subsequent research.

## 3. Discussion

The proliferation and differentiation of myocytes are crucial for skeletal muscle development, impacting meat quality and quantity in livestock. Understanding the regulatory mechanisms can improve meat quality and provide insights for therapeutic targets. Prior research found that *MYH7B* is related to the growth and development of sheep [29]. Additionally, it was found that knocking down *SOX6* upregulated slow skeletal muscle protein genes and downregulated fast skeletal muscle protein genes, indicating that *MYH7B* and *RUNX2* are possibly direct targets of *SOX6* affecting the muscle development of chickens [30]. Expression profiles indicate that *MYH7B* is involved in the muscle development of New Zealand white and Fujian yellow rabbits [31]. Relevant studies have shown that mice lacking the transcription factor *Nfix* experience delayed regeneration and convert to oxidative fiber types [32]. So far, *Nfix* has been mainly studied in mice. The lack of understanding of its specific functions and signaling mechanisms in sheep skeletal muscle cells underscores the need for further research in this area.

Our research, using CCK-8 assays, immunofluorescence staining, Western blotting, and qRT-PCR, demonstrated that *Nfix* promotes the proliferation and differentiation of skeletal muscle cells and regulates *MSTN* expression. *MyoD* plays a dual role in mediating the proliferation and differentiation of myogenic cells: it is induced by *Myf5*, activating cyclins and *CDKs*, leading to myogenic cell proliferation, and it activates *CKI*, inducing cell cycle arrest and promoting myogenic differentiation. Additionally, *MyoD* is upregulated by *RB*, which promotes the expression of *cyclins/CDKs* and *CKIs*, mediating cell proliferation and differentiation. *RB* also regulates myoblast renewal or differentiation by upregulating *MyoD* or downregulating *cyclins* and *CDKs* [33]. Using CRISPR/Cas9 gene editing technology, it was found that knocking out *Myomaker* and *Myomerger* separately significantly inhibits myocyte fusion [34]. In cases of *Myf5* and *MyoD* double mutations, *MRF4* can maintain the identity of skeletal muscle and partially compensate for the functional loss of *MyoD*. *MRF4* promotes the formation and development of muscle fibers by activating downstream myogenesis-related genes [35]. *MSTN* limits muscle size by inhibiting the proliferation of muscle stem cells (satellite cells) and promoting their apoptosis [36]. *MSTN* knockout significantly reduces the proliferation rate of equine muscle satellite cells [37]. While *Nfib* is expressed in both lung interstitium and epithelium, mice lacking *Nfib* exhibit severe lung maturation defects and die at birth [38], and *Nfix*-deficient fetuses show disorganized sarcomerogenesis, likely due to delayed sarcomere assembly, though postnatal sarcomeres appear normal [39]. In summary, these results indicate that *Nfix* positively regulates myogenesis.

Summarily, *Nfix* positively regulates myogenesis. Our results indicated that *Nfix* overexpression upregulated *MyoD* and downregulated *MSTN* and *SMAD4*, suggesting its role in promoting differentiation. Conversely, *Nfix* knockout had opposite effects. Research has shown that in the Wnt/β-catenin signaling pathway, β-catenin can directly act on *MyoD*, promoting its binding to the E-box element and enhancing *MyoD’*s transcriptional activity. Since *MyoD* is crucial for muscle differentiation, this interaction promotes muscle development. Conversely, when β-catenin is absent or its interaction with *MyoD* is obstructed, the transcriptional activity of *MyoD* is suppressed [40]. Blocking the BMP signaling pathway in mice leads to a decrease in the number of skeletal muscle satellite cells, hindering muscle growth [41]. The TGF-β signaling pathway mainly inhibits myogenesis differentiation by suppressing the expression of key myogenic transcription factors such as *MyoD* and *myogenin*. This pathway also plays a significant role in muscle regeneration, fiber type conversion, and muscle diseases [42]. Specifically, TGF-β inhibits myogenesis differentiation by activating *Smad2/3* and other downstream signaling molecules. When *ActRIIB* binds with *MSTN*, it recruits and phosphorylates *Smad2/3*, activating downstream signaling pathways that ultimately inhibit muscle growth [43].

MicroRNAs (miRNAs) play a role in muscle development. Our results show that *Nfix* and *MSTN* share five common target miRNAs, with miR-423-5p playing a regulatory role in skeletal muscle cell differentiation. During muscle cell differentiation, the myogenic transcription factor *MyoD* upregulates miR-206 expression by binding to the miR-206 promoter, thus enhancing muscle cell differentiation [44]. In mouse C2C12 cells, miR-1a-3p, miR-206-3p, miR-24-3p, and miR-486-5p regulate the differentiation of skeletal muscle cells by targeting the 3′UTR region of the transcription factor *MRTF-A* [45]. miR-100-5p regulates skeletal muscle myogenesis through the Trib2/mTOR/S6K signaling pathway [46], while miRNA-127 enhances skeletal muscle cells proliferation and differentiation by targeting *S1PR3* [47]. Strongly expressed in pig skeletal muscle, miR-423-5p overexpression significantly reduces the expression of *MyoD* and myogenesis differentiation antigens [48]. During mouse embryo development, the high expression of *Cyclin E* in embryonic stem cells is due to the transcriptional activation of the transcription factor *Esrrb*, working in synergy with its negative regulator miR-15a [49].

Although our study has preliminarily demonstrated that *Nfix* influences the proliferation and differentiation of myoblasts, we acknowledge several limitations. One limitation is the relatively small sample, which might constrain the generalizability of the findings. Another limitation is the need for further validation of the interactive regulatory relationship between *Nfix*, miRNA, and *MSTN*. Clarifying the primary pathway through which *Nfix* inhibits *MSTN* to regulate muscle development is essential. Additionally, exploring other potential roles of *Nfix* could enrich our understanding. Addressing these limitations will enhance the credibility of our research and offer a more comprehensive perspective.

## 4. Methods

### 4.1. Animal Sample

We chose five healthy adult Chinese merino sheep from each period, all exhibiting good physical health. Samples were collected on the 35th (D35), 40th (D40), and 45th (D45) days of pregnancy for transcriptomic analysis. We also collected the longissimus dorsi muscle from a healthy Chinese merino sheep fetus at 135 days of gestation. To isolate mature muscle cells from the sheep, we used a two-step enzyme digestion method with yype I collagenase and trypsin. All samples were provided by the sheep farm of the Biotechnology Research Institute of Xinjiang Academy of Animal Science.

This study was conducted in accordance with the ethical guidelines of the Institutional Animal Care and Use Committee of the Xinjiang Academy of Animal Science, Urumqi, China (approval 2016ZX08010-004-009). The Chinese merino fine-wool sheep used in this research were maintained in optimal conditions at the Research Base of Sheep Breeding of the Xinjiang Academy of Animal Science. Surgical procedures were performed under strict aseptic protocols to minimize animal suffering. All animal handling and experimental procedures were carried out with a focus on ethical standards and the welfare of the animals involved, ensuring their humane treatment throughout the study.

### 4.2. Cell Culture and Reagents

The sheep myoblast cells (Culture Collection of Institute of Biotechnology, Xinjiang Academy of Animal Science, Xingjiang, China) were digested at 37 °C under constant shaking with a solution containing collagenase I (100 mg/mL, Sigma-Aldrich, St. Louis, MO, USA). The cells were then cultured in Dulbecco’s modified Eagle’s medium (DMEM, Gibco, Grand Island, NY, USA) containing 20% fetal bovine serum (FBS, Gibco, Grand Island, NY, USA) and 1% penicillin–streptomycin. For differentiation, samples were switched to DMEM supplemented with 2% horse serum (Gibco, Grand Island, NY, USA). The medium was changed every alternate day. The incubation environment was set to 37 °C and 5%CO_2_.

### 4.3. Vector Construction and Transfection

Sheep *Nfix* complementary DNA (cDNA) and Exon 2 (GenBank accession number XM_027969535.2) were amplified by polymerase chain reaction (PCR).

An HA sequence was added at the 3′ end for the detection of *Nfix* expressed protein (HA tag: TACCCATACGACGTCCCAGACTACGCT). These were then cloned into the Plex-mcs and lentiCRISPRv2-Puro vector from our laboratory. To investigate the effects of sheep *Nfix* on myoblasts, 293T cells were transiently transfected with *Nfix* overexpression and knockout plasmids. The packaging plasmids (psPAX2 and pMD2.G) were co-transfected into 293T cells for lentivirus packaging using the liposome transfection method. The transfection was performed using Lipofectamine 3000 (Invitrogen, Carlsbad, CA, USA) following the manufacturer’s instructions. The filtered supernatant was used immediately to infect the target cells. Primer sequences are listed in Table 2.

### 4.4. Cell Proliferation Assay

The proliferation of the control and overexpression groups or knockout groups were analyzed using a microscope (Leica, Heidelberg, Germany). Cell proliferation was quantified using the Cell Counting Kit 8 (CCK-8) assay, performed as previously described. Briefly, negative control (K) and transfected cells were incubated with 10% CCK-8 solution (Beyotime Biotechnology, Shanghai, China) at 37 °C for 1 h in the dark. The absorbance was then measured at 450 nm to determine the proliferation ability.

### 4.5. Immunofluorescence Staining

Cells were fixed in 4% paraformaldehyde after washing with PBS. The fixed cells were then permeabilized with 0.5% Triton X-100 and blocked for 20 min. After that, 5% BSA was added and the cells were sealed for 30 min, followed by washing with PBS three times, each for 5 min. The cells were then incubated overnight with mouse desmin antibody (22170110; Sigma, St. Louis, MO, USA, 1:150) at 4 degrees Celsius. Finally, the cells were incubated with FITC-labeled goat anti-mouse secondary antibodies (1:100) (ZF-0313; ZSGB-BIO, Beijing, China, 1:400) for 30 min at room temperature (about 25 degrees Celsius) and with 5 ug/mL DAPI (4′,6-diamidino-2-phenylindole) for 5 min. Digital images were captured using a fluorescence microscope (Leica image analysis system, model Q500MC).

### 4.6. Protein Extraction, Denaturation, and Expression Analysis

Transfected cells were lysed in RIPA buffer with 1% PMSF, and the protein was loaded onto an SDS-PAGE gel and transferred onto a PVDF membrane. Non-specific binding was blocked with 5% non-fat milk in Tris-buffered saline with Tween 20 for 2 h. Then, the proteins were incubated overnight with β-actin (GB1500, 11:1500, Servicebio, Wuhan, Hubei, China) as the internal reference, MSTN (TD13273, 1:1000, Abmart, Shanghai, China) as the target, and monoclonal anti-HA (H3663, 1:1500, Sigma, St. Louis, MO, USA) as a tag at 4 °C. The blots were subsequently incubated with goat anti-mouse (A0216, Beyotime, Haimen, Jiangsu, China) and goat anti-rabbit (A208, Beyotime). ECL substrates were used to visualize the signals (Beyotime, Haimen, Jiangsu, China, P0018A). ImageJ software (version 1.53e) was used to conduct a quantitative analysis of the Western blotting results according to the gray value of the strip.

### 4.7. RNA Extraction, cDNA Synthesis, and Expression Analysis

Total RNA from cells was extracted using TRIzol reagent (Invitrogen, Carlsbad, CA, USA) according to the manufacturer’s instructions and then reverse-transcribed into cDNA using a transcriptase kit (TIANGEN, Beijing, China). For expression analysis, quantitative real-time PCR (qRT-PCR) was carried out on a Bio-Rad PCR system using SYBR Green Master Mix (TIANGEN, Beijing, China) and gene-specific primers. GAPDH was used as an internal control. Fold changes in the indicated genes were analyzed using the 2^−∆∆CT^ method. Primer sequences are listed in Table 3.

### 4.8. Statistical Analysis

Results are expressed as the means ± standard error of the mean (SEM). Statistical differences between groups were determined using one-way ANOVA and two-way ANOVA [50], and a *p*-value < 0.05 was considered statistically significant (* *p* < 0.05, ** *p* < 0.01). GraphPad Prism 10.1.2 was used for data processing and graphing. To ensure the validity of the ANOVA results, we verified the assumptions of normality and homogeneity of variances using Shapiro–Wilk and Levene’s tests, respectively. Tukey’s HSD test was used for post hoc comparisons to identify significant differences between specific groups.

## Figures and Tables

**Table 1:** Top 10 potential genes with relatively high expression levels.

Gene Name	D45_FPKM	D40_FPKM	Log2 Fold Change	*p*-Value	padj
*COL1A1*	1380.63	389.61	2.10	2.77 × 10^−29^	2.46 × 10^−26^
*KERA*	91.81	29.50	1.93	3.51 × 10^−23^	9.24 × 10^−21^
*DLC1*	39.28	33.85	1.57	5.94 × 10^−28^	4.44 × 10^−25^
*MYH7B*	34.62	10.98	1.88	3.43 × 10^−18^	3.62 × 10^−16^
*AEBP1*	21.77	8.37	1.66	1.71 × 10^−17^	1.57 × 10^−15^
*NFIX*	20.28	9.16	1.62	2.26 × 10^−13^	8.88 × 10^−12^
*HIF3A*	19.43	6.28	1.92	2.36 × 10^−19^	3.09 × 10^−17^
*SSC5D*	12.67	5.17	1.60	5.37 × 10^−15^	3.04 × 10^−13^
*RYR3*	10.95	4.30	1.59	6.89 × 10^−16^	4.52 × 10^−14^
*CAMTA2*	10.80	4.62	1.53	2.38 × 10^−14^	1.17 × 10^−12^

**Table 2:** Nucleotide sequence of primers related to cloning the *Nfix*-sgRNA/Plex-*Nfix* expression vector.

Gene	Primer Sequence (5′→3′)
*Nfix*-F	ccgactctactagaggatccactagtgccaccatgtactccccgtactgcctcacccag
*Nfix*-R	gacgcgtcgggccctctagactcgagtcaagcgtagtctgggacgtcgtatgggtagag
*Nfix*-sgRNA-CF1	**Caccg**ACTTGCGCTTCCGCGCCTGC
*Nfix*-sgRNA-CR1	**aaac**GCAGGCGCGGAAGCGCAAGT**c**
*Nfix*-sgRNA-CF2	**Caccg**GGGGGGCTTCTTGCCCGTGA
*Nfix*-sgRNA-CR2	**aaac**TCACGGGCAAGAAGCCCCCC**c**
*Nfix*-sgRNA-CF3	**Caccg**ACCAGAAGGGCAAGATCCGG
*Nfix*-sgRNA-CR3	**aaac**CCGGATCTTGCCCTTCTGGT**c**

Note: The text in bold represents the BsmB I enzyme cut site.

**Table 3:** Sequences of real-time fluorescent quantitative PCR-related primers.

Gene	Primer Sequence (5′→3′)	Annealing Temperature (°C)	Product Length (bp)
*MSTN*	F:GTGATGAGCACTCCACAGAA	60	118
	R:CCAGAGCAGTAATTGGCCTT		
*SMAD4*	F:ACACACCTAATTTGCCTCAC	60	124
	R:TTAGAAATAGGAGGCTGGAA		
*MyoD*	F:CGACTCGGACGCTTCCAGT	60	105
	R:TAAGCGCGGTCGTAGCAGTT		
*Nfix*	F:GCCCGAGATCAAGCAGAAGTG	60	176
	R:CCTGGCGAAGGCAGTCAATCC		
*GAPDH*	F:GAAGGTCGGAGTGAACGGATT	60	217
	R:GGTCATAAGTCCCTCCACGAT		

## Data Availability

The original contributions presented in the study are included in the article/Supplementary Materials. Further inquiries can be directed to the corresponding author.

## Supplementary Materials

The following supporting information can be downloaded at https://www.mdpi.com/article/10.3390/ijms252211988/s1.
