# Supplementary material for: Regulatory Role of Nfix Gene in Sheep Skeletal Muscle Cell Development and Its Interaction Mechanism with MSTN
Source: Int J Mol Sci. 2024 Nov 8;25(22):11988. doi: 10.3390/ijms252211988 (PMC11593348; doi:10.3390/ijms252211988)

NFIXT1

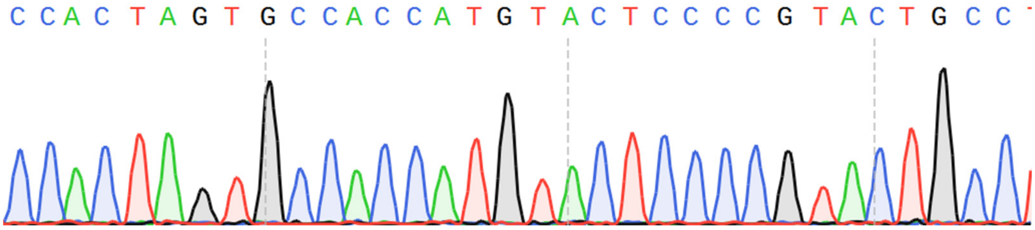

NFIX-T1

Target sequence: (gray is the clone site; Kozak sequence in blue; HA label in yellow)

ACTAGTGCCACCATGTACTCCCCGTACTGCCTCACCCAGGATGAGTTCCACCCGTTCATCGAGGCGCTG
CTGCCTCACGTCCGCGCCTTCTCCTACACCTGGTTCAACCTGCAGGCGCGGAAGCGCAAGTACTTCAAG
AAGCACGAGAAGCGGATGTCGAAGGACGAGGAGCGGGCGGTGAAGGACGAGCTGCTGGGCGAGAAGCCC
GAGATCAAGCAGAAGTGGGCATCCCGGCTGCTGGCCAAGCTGCGCAAAGACATCCGGCCCGAGTTCCGG
GAGGACTTTGTGCTGACCATCACGGGCAAGAAGCCCCCCTGCTGTGTGCTCTCCAACCCCGACCAGAAG
GGCAAGATCCGGCGGATTGACTGCCTTCGCCAGGCCGACAAGGTGTGGAGGCTGGACCTGGTCATGGTG
ATTTTGTTTAAGGGGATCCCCCTGGAAAGTACTGACGGGGAGCGGCTCTACAAGTCGCCCCAGTGCTCG
AACCCCGGCCTGTGCGTGCAGCCACATCACATTGGAGTCACAATCAAAGAACTGGATCTTTATCTGGCT
TACTTTGTCCACACTCCGGAATCCGGACAATCAGATAGTTCAAACCAGCAAGGAGATGCGGACATCAAA
CCACTGCCCAACGGGCACTTAAGTTTCCAGGACTGTTTTGTGACTTCCGGGGTCTGGAATGTGACGGAG
CTGGTGAGAGTATCACAGACTCCTGTTGCAACAGCATCAGGGCCCAACTTCTCGCTGGCAGACCTGGAG
AGTCCCAGCTACTACAATATCAACCAGGTGACCCTGGGGCGGCGGTCCATCACCTCCCCTCCCTCCACC
AGCACCACCAAGCGCCCCAAGTCCATCGATGACAGTGAGATGGAGAGCCCTGTCGACGACGTCTTCTAT
CCTGGCACAGGCCGCTCCCCGGCAGCTGGCAGCAGCCAGTCCAGTGGTTGGCCCAACGATGTGGATGCA
GGCCCGGCTTCCCTAAAGAAGTCAGGAAAGCTGGACTTCTGCAGTGCCCTCTCCTCTCAGGGCAGCTCC
CCGCGCATGGCTTTCACTCACCACCCGCTGCCTGTGCTTGCTGGAGTCAGACCAGGGAGCCCCCGGGCC
ACGGCGTCAGCGCTGCACTTCCCCTCCACATCCATCATCCAGCAGTCGAGCCCGTACTTCACGCACCCG
ACCATCCGCTACCACCACCACCACGGGCAGGACTCGCTGAAGGAGTTCGTGCAGTTCGTGTGCTCGGAC
GGCTCGGGCCAGGCCACCGGACAGCCCAACGGTAGCGGCCAGGGCAAAGTCCCGGGGTCATTTTTGCTA
CCACCGCCGCCTCCAGTGGCCAGACCTGTGCCCCTTCCTATGCCTGATTCCAAATCCACCAGCACTGCC
CCAGATGGCGCCGCCTTGACTCCTCCGTCACCTTCATTCGCAACGACAGGCGCCTCCTCTGCCAACCGG
TTTGTCAGCATCGGACCCCGGGACGGCAACTTTCTGAACATCCCACAGCAGTCTCAGTCCTGGTTCCTC
TACCCATACGACGTCCCAGACTACGCTTGACTCGAG

NFIXT3

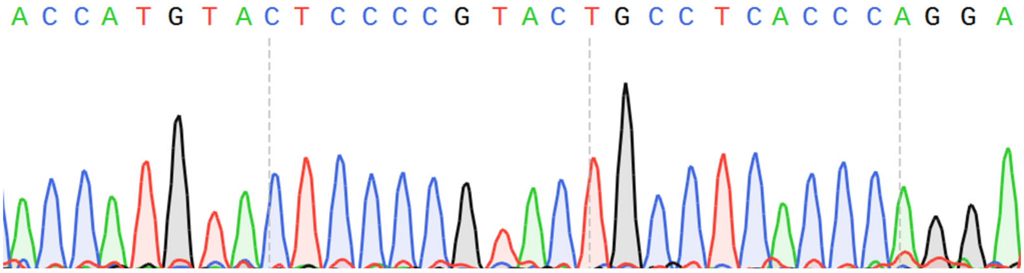

ACTAGTGCCACCATGTACTCCCCGTACTGCCTCACCCAGGATGAGTTCCACCCGTTCATCGAGGCGCTG
CTGCCTCACGTCCGCGCCTTCTCCTACACCTGGTTCAACCTGCAGGCGCGGAAGCGCAAGTACTTCAAG
AAGCACGAGAAGCGGATGTCGAAGGACGAGGAGCGGGCGGTGAAGGACGAGCTGCTGGGCGAGAAGCCC

GAGATCAAGCAGAAGTGGGCATCCCGGCTGCTGGCCAAGCTGCGCAAAGACATCCGGCCCGAGTTCCGG
GAGGACTTTGTGCTGACCATCACGGGCAAGAAGCCCCCCTGCTGTGTGCTCTCCAACCCCGACCAGAAG
GGCAAGATCCGGCGGATTGACTGCCTTCGCCAGGCCGACAAGGTGTGGAGGCTGGACCTGGTCATGGTG
ATTTTGTTTAAGGGGATCCCCCTGGAAAGTACTGACGGGGAGCGGCTCTACAAGTCGCCCCAGTGCTCG
AACCCCGGCCTGTGCGTGCAGCCACATCACATTGGAGTCACAATCAAAGAACTGGATCTTTATCTGGCT
TACTTTGTCCACACTCCGGAATCCGGACAATCAGATAGTTCAAACCAGCAAGGAGATGCGGACATCAAA
CCACTGCCCAACGGGCACTTAAGTTTCCAGGACTGTTTTGTGACTTCCGGGGTCTGGAATGTGACGGAG
CTGGTGAGAGTATCACAGACTCCTGTTGCAACAGCATCAGGGCCCAACTTCTCGCTGGCAGACCTGGAG
AGTCCCAGCTACTACAATATCAACCAGGTGACCCTGGGGCGGCGGTCCATCACCTCCCCTCCCTCCACC
AGCACCACCAAGCGCCCCAAGTCCATCGATGACAGTGAGATGGAGAGCCCTGTCGACGACGTCTTCTAT
CCTGGCACAGGCCGCTCCCCGGCAGCTGGCAGCAGCCAGTCCAGTGGTTGGCCCAACGATGTGGATGCA
GGGAGCCCCCGGGCCACGGCGTCAGCGCTGCACTTCCCCTCCACATCCATCATCCAGCAGTCGAGCCCG
TACTTCACGCACCCGACCATCCGCTACCACCACCACGGGCAGGACTCGCTGAAGGAGTTCGTGCAG
TTCGTGTGCTCGGACGGCTCGGGCCAGGCCACCGGACAGCCCAACGGTAGCGGCCAGGGCAAAGTCCCG
GGGTCATTTTTGCTACCACCGCCGCCTCCAGTGGCCAGACCTGTGCCCCTTCCTATGCCTGATTCCAAA
TCCACCAGCACTGCCCCAGATGGCGCCGCCTTGACTCCTCCGTCACCTTCATTCGCAACGACAGGCGCC
TCCTCTGCCAACCGGTTTGTCAGCATCGGACCCCGGGACGGCAACTTTCTGAACATCCCACAGCAGTCT
CAGTCCTGGTTCCTCTACCCATACGACGTCCCAGACTACGCTTGACTCGAG

Sg1

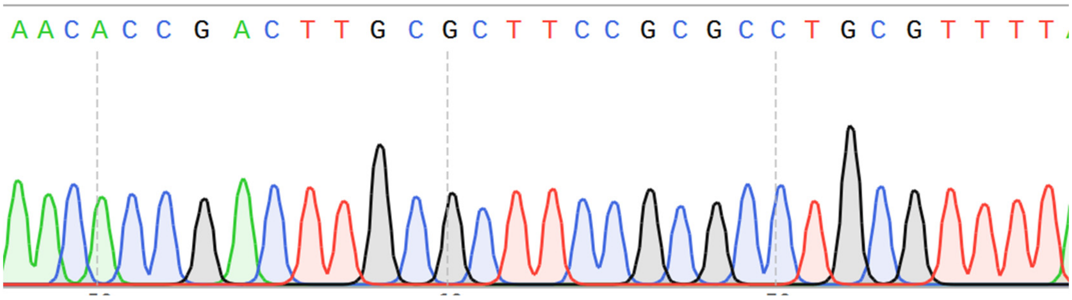

Sg2

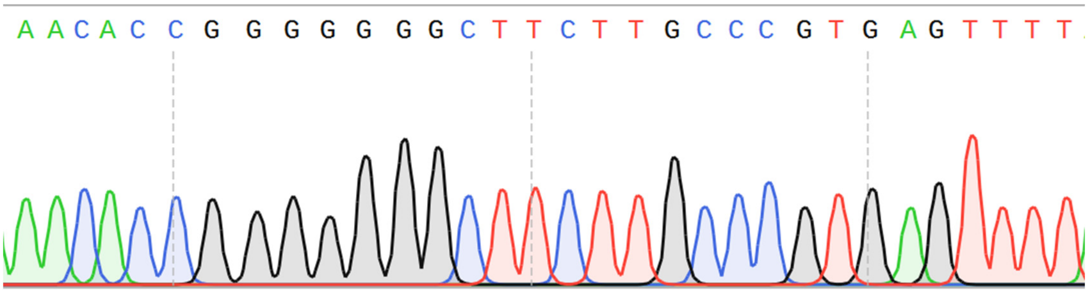

Sg3

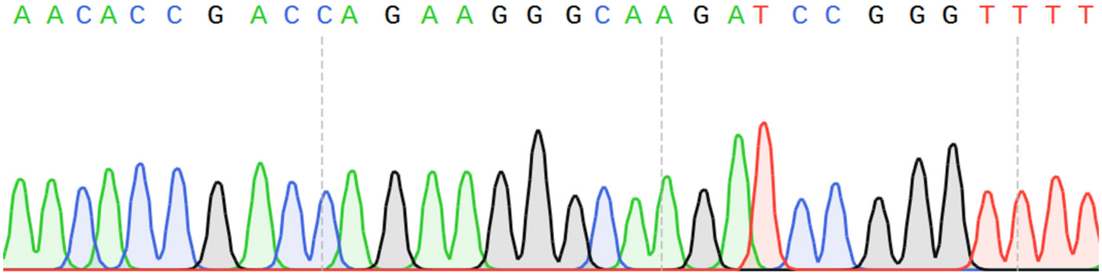

Supplement: Supplementary file 1 [file ijms-25-11988-s001.zip › Supplementary Materials S1.pdf]
